# Acute myocardial infarction in a young patient with Chikungunya: a case report

**DOI:** 10.1590/S1678-9946202567060

**Published:** 2025-08-25

**Authors:** João Gabriel Costa, Pedro Manuel Barros de Sousa, Marina Medeiros Orsi, Marcos Adriano Garcia Campos, Romullo José Costa Ataides, Joyce Santos Lages, Gyl Eanes Barros Silva

**Affiliations:** 1 Universidade Federal do Maranhão, Hospital Universitário, São Luís, Maranhão, Brazil; 2 Universidade Estadual Paulista, Hospital das Clínicas, Botucatu, São Paulo, Brazil; 3 Duke University, School of Medicine, Department of Emergency Medicine, Durham, North Carolina, USA; 4 Universidade de São Paulo, Hospital das Clínicas, São Paulo, São Paulo, Brazil

**Keywords:** Chikungunya virus, Acute myocardial infarction, Inflammation, Autopsy

## Abstract

Chikungunya virus (CHIKV) is globally distributed and transmitted by *Aedes* mosquitoes, with a mortality rate of 0.8/1,000 cases. The heart is the second most affected organ, with the osteoarticular system being the first. Cardiac involvement ranges from acute symptoms like myocarditis and exacerbation of pre-existing conditions to long-term complications such as dilated cardiomyopathy. While a direct association between CHIKV and acute myocardial infarction (AMI) is rare, systemic inflammation associated with chronic post-Chikungunya arthritis may destabilize atherosclerotic plaques, increasing AMI risk. This case report describes an AMI with non-obstructive coronary arteries in a previously healthy 24-year-old male infected with CHIKV. He presented low back pain, nausea, sweating, dyspnea, progressive leg edema, fever, and polyarticular pain in the knees and ankles. He was in critical condition upon admission, with decreased consciousness and hemodynamic instability, requiring transfer to the intensive care unit. He died 24 h later. Autopsy revealed a significantly enlarged heart, no visible atherosclerosis in the coronary arteries, and an extensive infarction in the interventricular septum. Histology showed coagulation necrosis, alveolar hemorrhage, and hepatic congestion. RT-PCR for CHIKV was detected in the lungs and heart tissues, while tests for other infectious diseases were negative. Studies highlight the role of mitochondrial antiviral signaling protein (MAVS) in protecting cardiac tissue from chronic CHIKV-related effects. Impaired MAVS signaling may enable continued viral replication, leading to myocarditis and vascular inflammation. Co-infection with dengue fever further increases the risk of cardiac complications. Postmortem analysis is essential to confirm CHIKV-related cardiac deaths and improve understanding and management of severe manifestations.

## INTRODUCTION

Chikungunya virus (CHIKV) is transmitted globally by arthropods of the genus *Aedes*. Although its mortality rate is notably lower than that of dengue virus (DENV)—approximately 0.8 per 1,000 cases^1^— CHIKV can cause persistent joint pain and exacerbate pre-existing conditions. Recent research has underscored an increased long-term mortality risk following CHIKV infection, particularly due to cardiovascular complications. A cohort study reported a significant rise in the risk of ischemic heart disease, with a relative risk of 3.66 (95%CI: 1.25–13.96) within 28 days after exposure^2^.

According to the Pan American Health Organization (PAHO), there has been a rise in Chikungunya cases in the Americas, with over 410,000 instances reported in 2023, compared to 271,176 cases in 2022^3^. Given the growing disease burden, increased vigilance is essential regarding secondary and less common effects among populations exposed to the virus.

Preliminary studies have already linked arboviral infections to heart disease^4^. Currently, the heart is considered the second most affected organ, behind only the osteoarticular system. Manifestations range from acute symptoms, such as palpitations and arrhythmia, to myocarditis and exacerbation of underlying conditions, as well as delayed complications such as dilated cardiomyopathy as a sequela for infection^5^. A literature review identified a correlation between cardiac involvement and genetic lineages from East/South/Central Africa (ECSA) and Asia. Additionally, a phylogenetic study performed in Brazil found that ECSA is circulating locally^6^.

CHIKV can cause myocarditis by penetrating myocytes and triggering an inflammatory response characterized by cell damage, necrosis, and features of hypersensitivity. However, the correlation between CHIKV and acute myocardial infarction (AMI) remains low^7^. A series of 610 atypical cases during an outbreak found only four patients with AMI after CHIKV infection^8^. Another study found that patients with post-CHIKV chronic arthritis are at increased risk of AMI due to systemic inflammation promoting destabilization of coronary atherosclerotic plaques^9^.

This case report aims to describe a fatal case of AMI with non-obstructive coronary arteries in a young patient infected by CHIKV and to discuss the possible mechanisms involved.

### ETHICS

This research was approved by the Ethics Committee of the Hospital Universitario da Universidade Federal do Maranhao (HU/UFMA), under process Nº 4.069.664.

## CASE REPORT

A previously healthy 24-year-old male patient from Sao Luis city, Maranhao State, in Northeastern Brazil, was admitted to the emergency department with a four-day history of low back pain, nausea, sweating, dyspnea, and progressive leg edema. He also reported febrile episodes and polyarticular pain in the knees and ankles during the course of the illness.

The patient was in critical condition upon admission, presenting with a reduced level of consciousness (Glasgow Coma Scale = 8) and hemodynamic instability. He required ventilatory support and the use of vasoactive drugs after admission to the intensive care unit. Laboratory tests identified refractory severe metabolic acidosis and acute renal dysfunction. Although emergency hemodialysis was indicated, it was initially withheld due to hemodynamic instability. Despite clinical support, the patient died within 24 h of admission.

The autopsy was performed seven hours after death. Examination revealed epistaxis and swelling in both lower limbs. Upon opening the thoracic cavity, a moderate amount of citrine-yellow pleural effusion was identified. The heart was enlarged (746 g) without noticeable atherosclerosis in the aorta or coronary arteries. The myocardium exhibited a sizeable irregular area in the interventricular septum, with a soft consistency and yellowish color, which was sampled for histological analysis. The lungs and liver showed parenchymal hemorrhagic spots, and the liver also showed signs of chronic passive congestion. Blood and tissue samples were collected to test for arboviruses due to the patient’s history of fever and evidence of hemorrhage.

**Figure 1:**
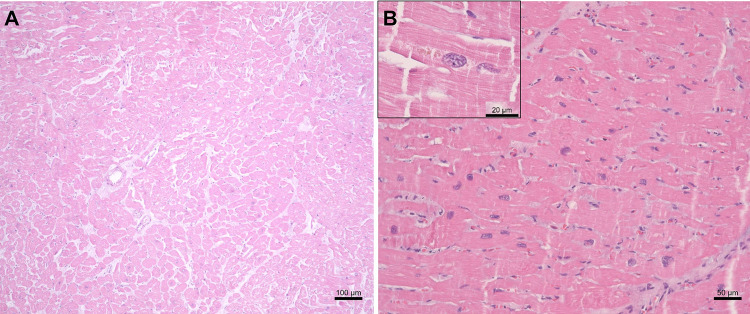
Cardiac tissue observed by light microscopy with hematoxylin and eosin staining: (A) Histopathological signs of acute myocardial infarction. Cardiac skeletal muscle fibers with eosinophilic cytoplasm and absence of viable nuclei, characteristic of coagulative necrosis; (B) Histopathological signs of dilated heart disease. Cardiomyocytes with enlarged and irregular nuclei, with a slight increase in the mononuclear inflammatory component in the connective tissue. Accumulation of lipofuscin in cardiac cells, a marker of cellular distress (inserted image).

Histological examination (Figure 1) revealed an extensive area of myocardial infarction with coagulation necrosis, alveolar hemorrhage, and hepatic congestion. Samples were tested using a standardized infectious disease panel from the Maranhao Central Public Health Laboratory. Tests were performed for HIV, leptospirosis, COVID-19, parvovirus, CMV, tuberculosis, and others. CHIKV was identified in lung and heart samples analyzed by RT-PCR, and had negative results for RT-PCR tests for DENV and Zika viruses.

## DISCUSSION

This case report describes a sudden cardiac deterioration observed in a patient with post-mortem findings indicative of alveolar hemorrhage and myocardial ischemia in the absence of atherosclerosis, associated with CHIKV infection. Such clinical presentation aligns with findings from a matched Brazilian cohort study, which reported an elevated risk of cardiovascular complications following CHIKV infection. The study highlights a critical post-infection window, during which cardiovascular monitoring is essential^2^.

CHIKV infection often presents with symptoms such as fever and severe joint pain, and recent studies have suggested a concerning association with cardiovascular issues^10^. However, CHIKV is highly symptomatic, with an attack rate of up to 70%^11^. Atypical cases—associated with a mortality rate of about 32.8%—often involve heart complications that require special attention. Polyarthralgia is recurrent in 30% to 40% of individuals and may persist for years. Atypical manifestations may include neurological and cardiac complications and effects on the gastrointestinal tract, liver, kidneys, skin, and hematologic cells^12^.

CHIKV infection begins when an infected *Aedes* mosquito bites a human, initiating viral replication of fibroblasts and macrophages at the inoculation site. The virus then enters the bloodstream and spreads to various tissues, including lymphoid organs, skin, muscles, and joints. CHIKV can also infect skeletal muscle satellite cells. The infection triggers a robust inflammatory response characterized by elevated levels of pro-inflammatory cytokines—such as interferon α, interferon γ, interleukin 6, and IP-10—and by immune cell infiltration into affected tissues^13^.

Delving deeper into the interaction between CHIKV and the host’s cardiovascular system, studies suggest that the virus can infiltrate cardiac fibroblasts, triggering a chain reaction of immune responses that may lead to heart-specific genetic mutations and contribute to cardiac pathologies^14^. This response is essential for viral clearance; however, when it is absent or impaired, individuals may become susceptible to persistent heart conditions^5^.

A recent study emphasized the importance of mitochondrial antiviral-signaling protein (MAVS) in protecting the heart from the chronic effects of CHIKV^15^. Inadequate MAVS signaling has been associated with sustained CHIKV replication in heart tissue, which can lead to myocarditis and chronic vascular inflammation—further supporting the CHIKV potential to cause long-term cardiovascular damage^15^.

Given these insights, MAVS signaling is essential for clearing CHIKV heart infection and preventing chronic vascular inflammation. Vigilant monitoring for heart health following CHIKV infection is therefore crucial. Healthcare providers must be aware of these risks, particularly for patients presenting relevant symptoms or with a history of exposure to the virus^16^.

Treating CHIKV infection with cardiac complications requires a multifaceted approach, including symptomatic treatment, careful cardiac monitoring, and standard treatments for myocardial infarction, pericarditis, or myocarditis. Integrating targeted symptomatic care with strategies to address the viral-mediated inflammatory response is necessary to optimize patient outcomes and prevent chronic cardiac sequelae^17^.

Co-infection with DENV and CHIKV has been reported, representing a global health concern^18^. While cardiac involvement such as myopericarditis has been observed in monoinfections, its occurrence in coinfected patients is rare, and few data are available on the overall cardiac effect of these coinfections. Current evidence is inconclusive regarding whether coinfection increases the severity of clinical manifestations compared to monoinfections and requires further investigation^18^. However, there is one case report of myocarditis after recent co-infection of CHIKV and DENV in a young, immunocompetent 28-year-old man^12^. Clinical manifestations of co-infections generally resemble severe forms seen in monoinfected individuals. Nonetheless, the combined risk of the severe outcomes associated with each virus—such as DENV hemorrhagic/neurological diseases and CHIKV-related chronic arthritis/cognitive disorders—raise significant concerns^19^. Due to similar initial symptoms that may lead to misdiagnosis and consequently to inappropriate treatment (such as the use of non-steroidal anti-inflammatory drugs in cases of DENV incorrectly diagnosed as CHIKV), it is crucial that both infections be evaluated during diagnosis^18^.

Autopsies in Chikungunya-related fatalities are vital for confirming the cause of death and for providing detailed insights into how the virus affects cardiac structures and reveal the mechanisms behind myocardial injuries^19^.

In the context of arbovirus infections, a growing body of research highlights the importance of both conventional autopsy (CA) and minimally invasive autopsy (MIA) for the understanding and surveillance of these diseases^20^. CA remains the gold standard for diagnosing the cause of death from DENV, contributing significantly to the detection of clinically undiagnosed cases and reducing underreporting. In Brazil, autopsies performed by the Death Verification Services (DVS) in the Ceara State, Northeast region, contributed to greater detection of deaths from DENV and CHIKV cases. However, the implementation of CA faces considerable challenges, including refusal of consent by family members, insufficient number of DVS units, lack of financial resources, and shortage of trained professionals. The refusal rate is particularly high in pediatric cases. In response to these barriers, MIA emerges as a promising alternative, being a more acceptable and less invasive technique for sample collection. Studies conducted in Ceara State, for instance, have aimed to establish protocols for the use of MIA in detecting deaths from arboviruses. These efforts have demonstrated that MIA can increase the system’s capacity to identify a greater number of suspicious deaths and improve detection sensitivity.

The initial experience in Ceara State confirmed deaths from CHIKV and DENV by using MIA^20^, and a specific case report demonstrated that this method was sufficient to confirm the diagnosis of severe dengue in a pediatric patient, with findings comparable to those obtained with CA. Although CA remains the gold standard, current research validates MIA as an effective and viable tool for the surveillance of deaths from arboviruses and other diseases of public health concern, especially when CA is not possible.

Thorough post-mortem examinations improve our understanding of the systemic effects of chikungunya, support epidemiological monitoring, and guide clinical interventions toward mitigating its severe manifestations. These examinations are critical for enhancing public health responses.

## CONCLUSION

The association between inadequate MAVS signaling and sustained CHIKV replication in the myocardium reveals the need to implement treatment strategies to protect the cardiac system in severe cases of chikungunya. Early monitoring and modulation of the inflammatory response are essential to prevent permanent cardiac sequelae. In fatal cases, autopsy is fundamental to understand the mechanisms involved in cardiac lesions and exclude differential diagnosis. It also facilitates the systemic effects of CHIKV, supports epidemiological monitoring, and guides targeted clinical interventions for severe disease manifestations. This case contributed to the growing body of Brazilian research on cardiovascular risk following CHIKV infection, enabling comparison with other documented fatalities and highlighting the relevance of early surveillance techniques to improve clinical management.
